# The Optimal Cutoff Value of Z-scores Enhances the Judgment Accuracy of Noninvasive Prenatal Screening

**DOI:** 10.3389/fgene.2021.690063

**Published:** 2021-07-21

**Authors:** Lingna Zhou, Bin Zhang, Jianbing Liu, Ye Shi, Jing Wang, Bin Yu

**Affiliations:** Changzhou Maternity and Child Health Care Hospital Affiliated to Nanjing Medical University, Changzhou, China

**Keywords:** noninvasive prenatal screening, Z-scores, prenatal screening, prenatal diagnosis, positive predictive value, false discovery rate

## Abstract

**Objective:**

To evaluate the accuracy of Z-scores of noninvasive prenatal screening (NIPS) in predicting 21, 18 trisomy, and X chromosome aneuploidy.

**Methods:**

A total of 39,310 prenatal women were recruited for NIPS from September 2015 to September 2020. Interventional prenatal diagnosis was applied to verify the diagnosis of NIPS-positive results. Logistic regression analysis was employed to relate the Z-scores to the positive predictive value (PPV) of NIPS-positive results. Using receiver operating characteristic (ROC) curves, we calculated the optimal cutoff value of Z-scores to predict fetal chromosome aneuploidy. According to the cutoff value, NIPS-positive results were divided into the medium Z-value (MZ) and high Z-value (HZ) groups, and PPV was calculated to access the accuracy of Z-scores.

**Results:**

A total of 288 effective values of Z-scores were used as the final data set. The logistics regression analysis revealed that Z-scores were significantly associated with true-positive results for 21 trisomy (T21) and 18 trisomy (T18) (*P* < 0.05), whereas the same was not observed for X chromosome aneuploids (*P* > 0.05). The optimal cutoff value of the Z-score for T21, T18, XO, XXX, and XXY indicated by ROC curve analysis were 5.79, 6.05, −9.56, 5.89, and 4.47, and the area under the curve (AUC) were 0.89, 0.80, 0.48, 0.42, and 0.45, respectively. PPV in the HZ group was higher than that in the MZ group, and the application of the cutoff value reduced the false discovery rate (FDR), which was only 2.9% in the HZ group compared with 61.1% in the MZ group for T21 and T18. The difference in total PPV between the MZ and HZ groups for X chromosome aneuploids was statistically significant. Moreover, the PPV for XXX and XXY seemed to increase with Z-scores but not for XO.

**Conclusion:**

The Z-score is helpful for the accurate judgment of NIPS results and for clinical prenatal counseling. Especially for T21 and T18, Z-scores have an excellent clinical association, which is superior to that seen with X chromosome aneuploids. In addition, using Z-scores to judge NIPS results offers a certain reference value for XXX and XXY but not for XO.

## Introduction

Prenatal screening and diagnosis are the most important strategies to prevent birth defects. After cell-free fetal DNA (cf-DNA) was discovered in 1997 (Lo et al., 1997), noninvasive prenatal screening (NIPS) has been rapidly developed and widely used all over the world (Lo et al., 2010). As we all know, NIPS is routinely used to screen for fetal autosomal aneuploidy, including trisomy 21 (T21), trisomy 18 (T18), and trisomy 13 (T13) with high accuracy and specificity (Xue et al., 2019). NIPS also enables screening for sex chromosome aneuploidies (SCAs), such as Turner (45, X), triple X (47, XXX), and Klinefelter syndromes (47, XXY) (Kornman et al., 2018). Recently, some reports also showed that it could contribute to the detection of fetal microdeletions, duplications, and single-gene diseases, such as DiGeorge syndrome (22q11 deletion) (Advani et al., 2017), Duchenne muscular dystrophy (Brison et al., 2019), and autosomal recessive nonsyndromic hearing loss (Han et al., 2017). Although NIPS is reported to be highly accurate for fetal T21, T18, and T13, especially with the development of high-throughput sequencing technology, a small percentage of women still have false-positive and false-negative results. Moreover, the high rate of false-positive results is the biggest problem in NIPS with the screening of SCAs and microdeletion and microduplication syndromes.

For most genome-wide methods, the result of NIPS for an individual woman is calculated as a Z-score, in which the individual sample is compared with a control group of normal (diploid) samples (Tian et al., 2018). Z-score (±3) is commonly used as the risk threshold to judge the fetal aneuploidy status in NIPS (Budis et al., 2019). Recently, in a study that employed the ion proton semiconductor sequencing platform, Z-scores of T21/T18/T13 were shown to be closely related to the accuracy of NIPS, and false-positive rates were higher when Z-scores at 3 ≤ Z < 5 compared with positive NIPS results at 5 ≤ Z < 9 and Z ≥ 9 (Tian et al., 2018). Another study showed that, under the BGISEQ-500 sequencing platform, the increase of Z-scores increased the likelihood of NIPS results of T21/T18/T13 being accurate (Junhui et al., 2021). However, these data are not enough to assess the accuracy of Z-scores of NIPS because different sequencing platforms have different testing procedures and classification algorithms of Z-scores. Furthermore, data on the evaluation of the accuracy of Z-scores of NIPS for other chromosome aneuploidies are not available.

In this study, NIPS was performed using the Illumina Next-Seq CN500 platform. We evaluated the performance of Z-scores of NIPS in the last 5 years based on logistic regression and receiver operating characteristic (ROC) analysis. We hope that this study can improve the assessment of Z-score accuracy of NIPS and be helpful to doctors in clinical counseling.

## Materials and Methods

### Subjects

From September 2015 to September 2020, 39,310 pregnant women who came to the Changzhou Maternity and Child Health Care Hospital, affiliated to the Nanjing Medical University, for prenatal screening and diagnosis were recruited for this study. They were 18–49 years old and were between 12^+0^ and 28^+0^ weeks of gestation. After informed consent, they volunteered to be tested by NIPS.

This study was approved by the Ethics Review Committee of the Changzhou Maternity and Child Health Care Hospital, affiliated to the Nanjing Medical University (No. 201501).

### Noninvasive Prenatal Screening

Eight milliliters of maternal blood were collected, and the plasma was centrifuged at 1,600 × *g* for 10 min at 4°C. Total cf-DNA was extracted from 1.2 mL of plasma using a nucleic acid extraction kit from Berry Genomics Co. Ltd., following the product protocol. DNA libraries were constructed using 40.5 μL of purified cf-DNA following the manufacturer’s protocol (Berry Genomics Co. Ltd., Beijing, China). DNA sequencing was performed using the Illumina Next-Seq CN500 platform according to the manufacturer’s instructions. The sequences of each sample which mapped to each chromosome were counted, and the GC content was calculated. Normalized chromosome representation and CG correction were used to generate a Z-score (Guseh, 2020). The fetal aneuploidy status was determined by Z-scores, and −3 < Z < 3 was the normal range.

### Prenatal Diagnosis

All women with NIPS-positive results were recalled and received genetic counseling. On a voluntary basis, they accepted prenatal diagnosis by amniocentesis after informed consent. Clinicians and pregnant women selected amniotic fluid karyotype analysis and/or chromosome microarray analysis according to their NIPS results. Cytogenetic analyses were performed using the GSL-120 instrument (Leica Biosystems Richmond, Inc.) and Cyto-Vision Automated Cytogenetics Platform software by two technicians independently. Chromosome microarray analysis was done using the Affymetrix Cyto-Scan 750 k chips and Chromosome Analysis Suite ver. 3.0 (ChAs) software. The detailed technical procedures were both reported previously (Yu et al., 2017; Wang et al., 2019).

### Statistical Analysis

All data were entered into Excel 2010 (Microsoft, Inc., Redmond, WA, United States). Statistical analyses were performed using the statistical software SPSS version 22.0 (SPSS, Inc., Chicago, IL, United States). The Z-scores were presented as the median with 25th and 75th percentiles (P25 P75) and analyzed using a nonparametric test. Logistic regression analyses were applied to relate the Z-scores to the positive predictive value (PPV) of positive NIPS results. ROC curves were calculated to assess the optimal cutoff value of Z-scores to predict the fetal chromosome aneuploidy. The area under the curve (AUC) quantifies the ability of the test to distinguish between normal fetus and fetal aneuploidy. Chi-squared test or Fisher exact tests were employed to compare differences for continuous variable; *P* < 0.05 was considered statistically significant.

## Results

### General Results

Among the 39,310 women who underwent the NIPS test, 364 tested positive for T21/T18/T13 and X chromosome aneuploidy. Their basic characteristics and general results were shown in Table 1. There were no significant differences in maternal age, gestational week, and body mass index (BMI) among the groups, which would reduce the interference factors in the Z-score assessment analysis. All women with NIPS-positive results were recalled and received genetic counseling again. Among them, 288 women accepted prenatal diagnosis, and 169 cases were confirmed as true positives, which comprised 106 cases for T21, 25 for T18, one for T13, and 37 for X chromosome aneuploidy. Thus, the effective values of Z-scores of the 288 women who accepted prenatal diagnosis were used as the final data set for assessing the Z-score accuracy of NIPS.

**TABLE 1:** Numbers and maternal characteristics of pregnancies with noninvasive prenatal screening (NIPS) positive results.

	**Numbers**					**Age**	**GW**	**BMI**
	**NIPS**+	**TP**	**FP**	**FN**	**Unverified**			
**Trisomy 21, 18, 13**								
T21	127	106	14	4	7	31.47 ± 5.43	17.35 ± 1.83	22.58 ± 2.79
T18	47	25	13	0	9	32.32 ± 5.29	17.15 ± 1.59	22.52 ± 2.60
T13	19	1	12	0	6	31.79 ± 4.17	16.84 ± 1.30	21.22 ± 2.36
Total	193	132	39	4	22	31.71 ± 5.28	17.24 ± 1.72	21.41 ± 2.72
**X chromosome aneuploidy**								
XO	93	10	55	1	28	30.92 ± 4.25	17.48 ± 1.82	22.50 ± 3.91
XXX	33	10	13	0	10	32.16 ± 5.38	17.14 ± 2.01	23.16 ± 3.71
XXY	45	17	12	0	16	32.02 ± 5.16	17.13 ± 1.61	23.26 ± 4.85
Total	171	37	80	1	54	31.42 ± 5.03	17.32 ± 1.79	22.84 ± 3.92

*NIPS, noninvasive prenatal screening; NIPS+, NIPS positive result; TP, true positive; FP, false positive; FN, false negative.*

*GW, gestational week; BMI, body mass index.*

### Logistics Regression and ROC Curve Analysis of Z-scores

In Table 2, the binary logistics regression model analysis reveals that Z-scores of NIPS-positive results were significantly associated with true positive results (OR = 1.05, *P* = 0.000376). In particular, Z-scores were significantly associated with true-positive results for T21 (OR = 1.78, *P* = 0.000171) and T18 (OR = 1.67, *P* = 0.011336). As only one case of T13 was diagnosed, logistics regression analysis was not performed. In addition, there was no significant difference for X chromosome aneuploids.

**TABLE 2:** Logistic regression analysis of pregnancies with trisomy 21 (T21), trisomy 18 (T18), and X chromosome aneuploidy.

**NIPS positive**	***B***	**SE**	**Wald**	**OR**	**95% CI**	***P***
T21	0.578	0.154	14.13	1.783	1.319–2.411	<0.001
T18	0.512	0.202	6.412	1.668	1.123–2.479	0.011
X0	0.054	0.065	0.671	1.055	0.928–1.200	0.413
XXX	–0.07	0.046	2.264	0.933	0.852–1.021	0.132
XXY	–0.102	0.06	2.911	0.903	0.802–1.015	0.088
Total	0.05	0.013	15.568	1.051	1.025–1.077	<0.001

**B*, beta coefficients; SE, standard error; Wald, wald test; OR, odds ratio; CI, confidence interval.*

Then, we analyzed the distributional difference between Z-scores of true and false positive results of NIPS. As shown in Table 3, the median Z-scores of NIPS true and false positive results were 11.7 and 5.4, respectively, which were significantly different (*P* = 0.001760). In particular, the Z-scores for T18 had an equally significant difference (9.34 vs. 5.11, *P* = 0.002837). However, similar results were not seen in the group of X chromosome aneuploids. There was no significant difference between the median Z-scores of true and false positive results of XO, XXX, and XXY aneuploidies.

**TABLE 3:** Distribution of Z-scores and receiver operating characteristic (ROC) curve analysis of pregnancies with T21, 18, and X chromosome aneuploidy.

	**Median (P25, P75) of Z-scores**		**ROC**			
	**TP**	**FP**	**AUC**	**Optimal cutoff value**	**Sensitivity (%)**	**Specificity (%)**
**Trisomy 21, 18**						
T21	11.70(8.25–14.88)	5.42(3.69–6.07)^*a*^	0.89	5.79	93.4	78.57
T18	9.34(6.48–10.88)	5.11(3.84–6.37)^*a*^	0.8	6.05	80	76.92
**X chromosome aneuploidy**						
XO	−5.76(−7.69 to −3.93)	−4.86(−7.95 to −3.37)	0.48	−9.56	100	21.82
XXX	8.86(7.68–9.84)	31.09(5.26–62.45)	0.42	5.89	100	30.77
XXY	6.18(4.80–7.35)	21.49(4.28–45.56)	0.45	4.47	94.12	33.33

*TP, true positive; FP, false positive; ROC, receiver operating characteristic; AUC, area under the curve.*

*^*a*^Stand TP vs. FP, *P* < 0.05.*

**FIGURE 1:**
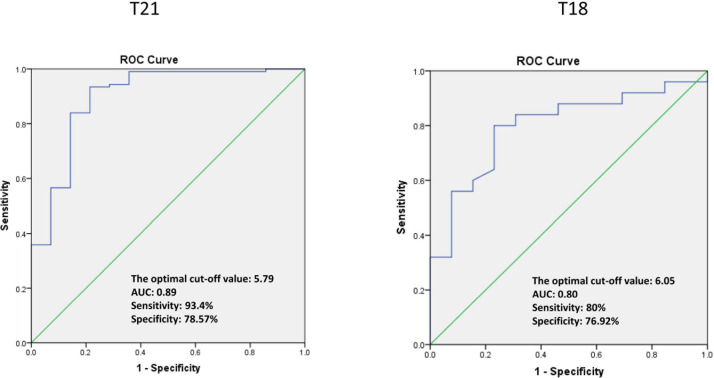
The ROC curve analysis of pregnancies with trisomy 21 and trisomy 18.

Afterward, ROC curve analysis was used to calculate the optimal cutoff value and the AUC for NIPS-positive results. The optimal cutoff value of Z-scores for T21 and T18 were 5.79 and 6.05, and the AUC were 0.89 and 0.80, respectively (Figure 1). In this study, the cutoff values revealed a sensitivity of 93.4 and 80.0% and a specificity of 78.6 and 76.9% for T21 and T18, respectively. On the other hand, the cutoff values were −9.56, 5.89, and 4.47 for XO, XXX, and XXY, respectively. The AUC were obviously lower than T21 and T18, which were 0.48, 0.42, and 0.45, respectively. Moreover, the sensitivity for XO, XXX, and XXY was high (100, 100, and 94.1%) although the specificity was low (21.8, 30.8, and 33.3%).

### Accuracy of Z-scores in Predicting T21 and T18 Aneuploidy

Based on the optimal cutoff value of Z-scores, the women with T21- and T18-positive results were divided into two groups: the medium Z-value (MZ) (3 ≤ Z < optimal cutoff value, MZ) and high Z-value (HZ) groups (Z > optimal cutoff value, HZ). As shown in Table 4, PPV in the HZ group was higher than in the MZ group. Moreover, there was a significant difference (*P* < 0.05) in PPV between the MZ group and all patients. Importantly, the application of the cutoff value reduced the false discovery rate (FDR) for T21, which was only 2.9% in the HZ group compared with 61.1% in the MZ group. Similarly, the difference in FDR for T18 was equally significant between two groups (13.0 vs. 66.7%, *P* < 0.05).

**TABLE 4:** The positive predictive value (PPV) and false discovery rate (FDR) performance of NIPS positive results with T21, 18.

	**Medium Z value group**			**High Z value group**			**All patients**		
	***n***	**PPV (%)**	**FDR (%)**	***n***	**PPV (%)**	**FDR (%)**	***n***	**PPV (%)**	**FDR (%)**
T21	18	38.89(7/18)	61.11(11/18)	102	97.06(99/102)^*a*^	2.94(3/102)^*a*^	120	88.33(106/120)^*b,c*^	11.67(14/120)^*b,c*^
T18	15	33.33(5/15)	66.67(10/15)	23	86.96(20/23)^*a*^	13.04(3/23)^*a*^	38	65.79(25/38)^*b*^	34.21(13/38)^*b*^
Total	33	36.36(12/33)	63.64(21/33)	125	95.2(119/125)^*a*^	4.8(6/125)^*a*^	158	82.91(131/158)^*b,c*^	17.09(27/158)^*b,c*^

*FP, false positive; FDR, false discovery rate; PPV, positive predictive value.*

*Medium Z value group: 3 ≤ Z < optimal cutoff value.*

*High Z value group: Z > optimal cutoff value.*

*^*a*^Stand for medium Z value group vs. high Z value group, *P* < 0.05.*

*^*b*^Stand for medium Z value group vs. all patients, *P* < 0.05.*

*^*c*^Stand for high Z value group vs. all patients, *P* < 0.05.*

Furthermore, to show more information about the distribution of Z-scores with T21 and T18, all patients were further classified based on Z-scores as 3 ≤ Z < 5, 5 ≤ Z < 10, and Z ≥ 10, and PPV were calculated. As shown in Table 5, the total PPV of T21 and T18 was 88.3% (106/120) and 66.8% (25/38), respectively. With the increase of Z-scores, an upward trend occurred in the PPV of T21 and T18. For example, when Z-scores were beyond 10 in T21, PPV could be 97.1%, which was significantly higher than 35.7% when Z-scores were lower than 5.

**TABLE 5:** The PPV performance of T21 and T18 in different classification of Z scores.

**Z scores**	**PPV (%) of T21**	**PPV (%) of T18**
3 ≤ Z < 5	35.71(5/14)	30.77(4/13)
5 ≤ Z < 10	92.11(35/38)	77.78(14/18)
Z ≥ 10	97.06(66/68)	100.00(7/7)
Total	88.33(106/120)	66.79(25/38)

*PPV, positive predictive value.*

### Accuracy of Z-scores in Predicting X Chromosome Aneuploidy

Most patients with SCAs are identified only after adolescence, especially as there are no significant features during initial follow-up in childhood. The accuracy of follow-up for SCAs was difficult to acquire, so we only observed one index (PPV) in this study. Based on the ROC curve analysis, Z-scores of X chromosome aneuploidy were also divided into medium (Z-score between optimal cutoff Z-score and ± 3, MZ) and HZ groups (Z-score > the absolute value of optimal cutoff value, HZ). As show in Table 6, there were significant differences between total PPV in the MZ and HZ groups (*P* = 0.000607) as well as between MZ group and all patients (*P* = 0.046059). Nevertheless, there was no significant difference between PPV in the MZ and HZ groups with XO, XXX, and XXY. Interestingly, the PPV seemed to increase with Z-scores for XXX and XXY, but not for XO.

**TABLE 6:** The PPV performance of XO, XXX, and XXY in different groups of Z-scores.

	**PPV (%) of medium Z value group**	**PPV (%) of high Z value group**	**PPV (%) of all patients**
XO	18.87(10/53)	0(0/12)	15.38(10/65)
XXX	0(0/4)	52.63(10/19)	43.48(10/23)
XXY	20(1/5)	66.67(16/24)	58.62(17/29)
Total	17.74(11/62)	47.27(26/55)^*a*^	31.62(37/117)^*b,c*^

*PPV, positive predictive rate.*

*Medium Z value group: patients with a Z-score between optimal cutoff Z-score and plus or minus three.*

*High Z value group: pregnancies with positive NIPS results at Z-score > the absolute value of optimal cutoff value.*

*^*a*^Stand for medium Z value group vs. high Z value group, *P* = 0.000607.*

*^*b*^Stand for medium Z value group vs. all patients, *P* = 0.046059.*

*^*c*^Stand for high Z value group vs. all patients, *P* = 0.046954.*

## Discussion

We believe that this study of Z-scores can improve the diagnostic predictive value of NIPS and be helpful to doctors in clinical counseling. The Z-score is the most commonly used method for the screening of fetal chromosome aneuploids. In addition, PPV depends not only on the sensitivity and specificity of the assay, but also on the prevalence of the disease, and unlike PPV, neither sensitivity nor specificity reflects the prevalence of a disorder in the population (Meck et al., 2015). In this study, the PPV is a population-based figure that reports the chance prior to prenatal testing that an abnormal NIPS test result is actually reflective of the karyotype of the fetus.

On the whole, logistic regression showed a significant association for NIPS-positive results between Z-scores and true-positive results, which suggested that the PPV performance of NIPS depended on the Z-scores. Then, based on the ROC analysis, we calculated the optimal cutoff value and AUC. According to our data, the AUC for T21 and T18 were larger, indicating a better ability to identify the optimal cutoff value of Z-scores for T21 and T18. Data for T21 and T18 were grouped based on the optimal cutoff value, and it was seen that the FDR in the HZ group was significantly lower than that in the MZ group, and the PPV in the HZ group was larger than that in the MZ group. Moreover, the classification of T21 and T18 Z-scores as 3 ≤ Z < 5, 5 ≤ Z < 10, and Z ≥ 10 verified that posteriori risk is effectively independent under all conditions for Z-scores above 6, and high posteriori risk for low *a priori* risk can only be reached at Z-scores > 5 (Sikkema-Raddatz et al., 2016). The application of using Z-scores as an indicator of laboratory judgment in NIPS had a good diagnostic predictive value for T21 and T18. However, the accuracy of Z-scores in predicting abnormal results for X chromosome aneuploidy needs more samples and data to improve.

Several reports (Hartwig et al., 2017; Chatron et al., 2019; Suzumori et al., 2021) showed that the accuracy of NIPS might be affected by confined placental mosaicism (CPM); vanishing twin, single, or multiple pregnancies; maternal tumors; concentration of cf-DNA; and chromosomal GC content. These effects were reflected in the accuracy of Z-scores. CPM occurs through a mitotic nondisjunction event or through aneuploidy rescue, which is the greatest obstacle in NIPS result confirmation (Zhang et al., 2015). Tian et al. (2018) also suggested that the occurrence of CPM may slightly elevate the Z-score for NIPS, higher than 3 but lower than 5. Moreover, maternal chromosome abnormalities may interfere with NIPS outcomes because approximately 85–90% of cf-DNA in maternal plasma is of maternal origin, and less than 15% is of fetal origin during the second trimester (McNamara et al., 2015). In this study, the values of Z-scores had increased in some false-positive cases for XXX and XXY, which may be associated with maternal X chromosome aneuploids. In our previous study (Zhang et al., 2020), we stated that the prediction of SCAs by NIPS may be interfered by maternal factors, and finding a way to deal with discordant SCA results caused by maternal sex chromosome abnormalities is the largest challenge remaining for NIPS.

It is widely known that one missed diagnosis due to a high Z-score cutoff value would be a disaster for the family involved. Therefore, clinicians should suggest at-risk pregnant women to go for further testing to confirm the diagnosis because NIPS is just defined as a screening technology rather than a diagnostic technology (Hartwig et al., 2017). The optimal cutoff values of Z-scores in this study are for clinician reference only and can be used in genetic counseling. The Z-score distribution of true and false positives of these chromosomes may be helpful for clinical counseling. Pregnant women whose NIPS results indicate risk of fetal abnormality should be treated differently depending on their choice of follow-up confirmation testing: (1) pregnant women are usually anxious when they are informed of the risk indicated by the results (Marcon et al., 2021). Pregnant women with a Z-score ≥ 3 and < the optimal cutoff value for T21 and T18 could be told about the possibility of false positive to alleviate anxiety. (2) A few at-risk pregnant women may refuse prenatal diagnosis. Clinicians must stress the importance of prenatal diagnosis in such cases.

Our study was based on the NIPS data of a relatively large population of 39,310 pregnant women. Unfortunately, some of the at-risk pregnant women refused prenatal diagnosis; hence, only one case of T13 was diagnosed. Therefore, logistics regression analysis was not undertaken, and the performance of Z-scores of T13 could not be analyzed. Moreover, a high rejection rate of prenatal diagnosis also occurred in pregnant women considered to be at risk of X chromosome aneuploidy. More samples and data are needed to improve the accuracy of Z-scores in predicting X chromosome aneuploidy. We know that different sequencing platforms have different testing procedures and classification algorithms of Z-scores. Multicenter cooperative research that employs the same technology platform may help in achieving a sounder conclusion.

In conclusion, Z-score value is helpful for the accurate judgment of NIPS results and for clinical prenatal counseling. Especially for T21 and T18 aneuploids, Z-scores have excellent clinical association that is superior to that for X chromosome aneuploids. In addition, using Z-scores to judge NIPS results offers a certain reference value for XXX and XXY although no such value is obtained for XO.

## Data Availability Statement

The datasets presented in this article are not readily available because Regulations on the management of human genetic resources in China. Requests to access the datasets should be directed to the corresponding author.

## Ethics Statement

The studies involving human participants were reviewed and approved by this study was approved by the Ethics Review Committee of Changzhou Maternity and the Child Health Care Hospital affiliated to Nanjing Medical University (NO. 201501). The patients/participants provided their written informed consent to participate in this study.

## Author Contributions

BY and JL carried out the assays and participated in the study design. LZ, BZ, JW, JL, and YS carried out the clinical consultations, laboratory tests, and performed the statistical analysis. BY and BZ conceived the study, participated in its design and coordination, and helped to draft the manuscript. All authors contributed to the article and approved the submitted version.

## Conflict of Interest

The authors declare that the research was conducted in the absence of any commercial or financial relationships that could be construed as a potential conflict of interest.

## Abbreviations

NIPS: noninvasive prenatal screening
PPV: positive predictive value
MZ: medium Z-value group
HZ: high Z-value group
ROC: receiver operating characteristic
AUC: area under the curve
FDR: false discovery rate
cf-DNA: cell-free DNA
T13: trisomy 13
T18: trisomy 18
T21: trisomy 21
SCA: sex chromosome aneuploidy.
